# Making the most of an admission: the safety and efficacy of higher caloric refeeding in hospitalised adolescents with restrictive eating disorders

**DOI:** 10.1186/2050-2974-3-S1-O66

**Published:** 2015-11-23

**Authors:** Elizabeth Parker, Sonia Faruquie, Gail Anderson, Linette Gomes, Danielle Hewitt, Andrew Kennedy, Christine Wearne, Michael Kohn, Simon Clarke

**Affiliations:** 1 Department of Dietetics and Nutrition, Westmead Hospital, Sydney, Australia; 2 Department of Adolescent Medicine, Westmead Hospital, Sydney, Australia; 3 Department of Medical Psychology, Westmead Hospital, Sydney, Australia

## Aim

This study examines weight gain and assesses complications associated with refeeding adolescent inpatients admitted with a restrictive eating disorder (ED) who were provided with caloric intakes above current recommendations.

## Methods

Patients admitted to an adolescent ED program for >48 hours were included in a 3-year retrospective chart review. The structured ‘rapid’ refeeding program mostly utilised initial nasogastric feeding, with routine phosphate prescription.

## Results

The mean (SD) age of the 184 adolescents was 16.7 years (0.9). Mean (SD) admission BMI was 16.9kg/m2 (2.3) and discharge BMI was 19.5kg/m2 (1.5). The mean (SD) starting caloric intake was 2523.6kcal/day (383.5) equating to 56kcal/kg (12). Most patients (87.5%) were treated with nasogastric tube feeding. Mean (SD) length of stay was 3.5 weeks (1.9) with a weekly weight gain of 2.1kg (0.9). No patients developed cardiac signs of refeeding syndrome or delirium; complications included peripheral oedema (3.8%), hypophosphatemia (1.1%), hypomagnesaemia (6%), and hypokalaemia (1.6%). Caloric prescription on admission was not associated with developing hypophosphatemia (p=0.15), hypokalaemia (p=0.40) and hypomagnesaemia (p=0.96).

## Conclusion

Results demonstrated the efficacy of treating adolescent inpatients with restrictive EDs safely with higher initial caloric intakes, resulting in rapid weight restoration without major refeeding complications; which challenges current conservative calorie prescriptions advocated in clinical guidelines.

